# Assessing the influence of cognitive response conflict on balance control: an event-related approach using response-aligned force-plate time series data

**DOI:** 10.1007/s00426-023-01809-9

**Published:** 2023-03-02

**Authors:** Leif Johannsen, Denise Nadine Stephan, Elisa Straub, Falko Döhring, Andrea Kiesel, Iring Koch, Hermann Müller

**Affiliations:** 1 Cognitive and Experimental Psychology, Institute of Psychology, RWTH Aachen University, Jaegerstr. 17/19, 52066 Aachen, Germany; 2 Department of Psychology, University of Freiburg, Freiburg, Germany; 3 Department of Sport Science, University of Gießen, Gießen, Germany

## Abstract

Process interference or sharing of attentional resources between cognitive tasks and balance control during upright standing has been well documented. Attentional costs increase with greater balancing demands of a balance activity, for example in standing compared to sitting. The traditional approach for analyzing balance control using posturography with a force plate integrates across relative long trial periods of up to several minutes, which blends any balance adjustments and cognitive operations within this period. In the present study, we pursued an event-related approach to assess if single cognitive operations resolving response selection conflict in the Simon task interfere with concurrent balance control in quiet standing. In addition to traditional outcome measures (response latency, error proportions) in the cognitive Simon task, we investigated the effect of spatial congruency on measures of sway control. We expected that conflict resolution in incongruent trials would alter short-term progression of sway control. Our results demonstrated the expected congruency effect on performance in the cognitive Simon task and the mediolateral variability of balance control within 150 ms before the onset of the manual response was reduced to a greater degree in incongruent compared to congruent trials. In addition, mediolateral variability before and after the manual response was generally reduced compared to variability following target presentation, where no effect of congruency was observed. Assuming that response conflict in incongruent conditions requires suppression of the incorrect response tendencies, our results may imply that mechanisms of cognitive conflict resolution may also carry over to intermittent balance control mechanisms in a direction-specific manner.

**Supplementary Information:**

The online version contains supplementary material available at 10.1007/s00426-023-01809-9.

## Introduction

The question of how the mind controls the body is not only relevant for philosophers but it has always been a core theme in cognitive psychology. The cognitivist notion that behaviorr is controlled by mental, “cognitive” processes now dominates in experimental psychology, but the mechanisms of how exactly cognitive and motor processes interact are still not well understood. Traditional stage models of information processing (e.g., Sternberg, 1969) have not suggested mutual interactions of cognitive and motor processes because the order of processing stages is seen as strictly serial and the stages themselves as encapsulated modules (see Sanders, 1998). As a consequence of this traditional separation, motor control processes remained somewhat neglected in mainstream experimental psychology (see Rosenbaum, 2005).

One prominent example in contemporary cognitive psychology is the “central bottleneck” model in attention research, of which a general assumption is that perceptual and motor processes need a “translation” by capacity-limited processes required by response decision and selection (see Pashler, 1998, for review). Notably, this translation is from stimulus-driven processing to motor processes so that the motor system is clearly at the “receiving end” of this processing chain. Yet, in cognitive psychology, a much closer interaction of cognitive and motor processes has been suggested by ideomotor approaches, which assume that motor action is activated by perceptual representations of anticipated sensory motor outcomes (e.g., Badets et al., 2016; Greenwald, 1970; Pfister, 2019; Prinz, 1997; Shin et al., 2010). A modern and generalized version of this integrated processing of perception and motor action postulates a more elemental coding in terms of perceptual and motor “features” that can be flexibly bound and unbound. These binding mechanisms, integration, and disintegration of basic feature codes thus create sequential, “episodic mini-building blocks” governing attention and action control (see Frings et al., 2020 for a recent review; Hommel, 2004). Importantly, the strict separation of cognitive and motor processes as well as the strict serial-stage logic is abandoned in these approaches, so that activation of motor codes could re-activate perceptual codes previously bound with them. This way, motor-related processes gain a much more prominent place in the respective cognitive models.

The present study was aimed at examining the cognitive-motor interactions in the control of body balance and posture (“balance control”). Balance control during quiet upright standing is an apparently static but actually highly dynamic activity caused by the interaction between body displacement through gravity pull and opposing muscle-produced torque. As long as the body’s Centre-of-Mass (CoM) ground projection remains within the limits of the support base, a standing posture can be considered stable. Successful balance control demands the integration of multiple sensory channels providing information about the body’s swaying motion relative to the support and other environmental impact (Peterka, 2002; Winter, 1995). In addition, the selection and execution of appropriate balance adjustments is a core demand of any balance activity.

Often, balance control while standing is assessed based on posturography by using force plates to measure body sway in terms of ground-reaction force dynamics (Duarte & Freitas, 2010). In line with Newton’s 3rd law of motion (reaction and interaction forces acting between two objects), modern force plates measure six components of the ground reaction force (three linear forces for each spatial direction; three angular moments around each spatial axis). The ground-reaction force is the sum of linear forces and angular moments that act in opposition to the forces and moments exerted by an individual’s muscular activity. The Centre-of-Pressure (CoP) is the location where the ground reaction force vector applies and represents an individual’s neuromuscular response to the dynamics of the CoM affected by the pull of gravity and all other forces acting on the body (Winter, 2005).

Variability of motor output in voluntary low-intensity plantar flexion and variability in body sway during quiet standing are related (Kouzaki & Shinohara, 2010) and motor output variability at the ankles determines balance stability in the sense that torque output at the ankle directly affects CoM displacement during quiet standing (Masani et al., 2003).

The stability of body sway in a specific situation can be analysed *****globally using several measures (Raymakers et al., 2005), for example variability measures such as the standard deviation or root mean square of CoP displacement or CoP velocity across a period of a certain duration (usually in the time range of 5–12 s or even up to 30 s, see for example Kang & Lipsitz, 2010; Kerr et al., 1985; VanderVelde et al., 2005; Vuillerme et al., 2000). In this context, the variability of body sway fluctuations is considered a combination of sensorimotor noise and fluctuations in central balance control mechanisms (Latash et al., 2002; van Emmerik & van Wegen, 2002). The torque around the ankle joint during plantarflexion, measured in terms of the angular moment around the mediolateral axis of force plate, controls the velocity and acceleration of the own body’s CoM in the anteroposterior direction according to the single inverted pendulum model (Horak & Nashner, 1986; Morasso et al., 2019). Complementarily, the moment around the anteroposterior axis of the force plate resembles the mediolateral torque exerted by the hip abductors/adductors (Winter et al., 1996). Here we decided against a combined measure such as CoP. Instead, we consider moment of force in the mediolateral and anteroposterior directions separately because both might show specific and distinguishable changes regarding velocity and acceleration of the own body’s COM.

In the domain of motor control research, a development can be seen that is in the complementary direction of motor-related processes in cognition. Here, it has been acknowledged that some issues of age-related motor deficits (i.e., increased risk of falls) are not so much motoric but more related to age-related cognitive changes in attentional control (Woollacott & Shumway-Cook, 2002). Such cognitive deficits could thus create particular problems with secondary-task load (e.g., in dual-tasking situations; see Boisgontier et al., 2013), suggesting that cognitive processes underlying divided attention can affect seemingly independent (“automatic”) motoric processes of balance control.

In this article, we focus on the interaction of cognitive control and balance control. We first briefly review findings on the influence of balance control demands on cognitive control. Then, we turn to the influence of attentional processes on balance control. Finally, we introduce a novel event-related methodology to assess the influence of cognitive processing conflict in a classic cognitive conflict task, the Simon task, on balance control parameters.

### Influence of balance control on cognitive control

Studies on the influence of balance control on discrete trial measures of attentional control are still scarce and the empirical findings are somewhat inconsistent. For example, Koch et al. (2003) compared performance when switching between two tasks, while lying or sitting. In this study participants performed two visual-spatial categorization tasks with manual keypress responses, in which a spatial target could appear in a 2 × 2 grid either at a left vs. right (horizontal task) or an upper vs. lower position (vertical task). The currently required task was pre-cued in each trial (Meiran, 1996). Please note that this setting also involves response conflict in terms of between-task interference. If, for example, “left” and “up” are mapped to the same response, then an upper left target would be “congruent,” whereas an upper right target would be “incongruent” because the two stimulus attributes would activate conflicting response options depending on the current task (i.e., horizontal vs. vertical categorization). The authors observed that a supine body position (i.e.**** lying) results in a generally increased reaction time (RT) level compared with a standard sitting posture, but there was no effect on more specific measures of selective attention, such as task switch costs (i.e., the performance difference between switching vs. repeating a task) and the congruency effect (see Kiesel et al., 2010; Koch et al., 2018; Monsell, 2003 for reviews). However, more recently, Stephan et al. (2018) used an auditory-manual version of such categorization tasks and compared task-switching performance in sitting vs. standing posture. They found no overall effect on RT or on switch costs but a specific increase of the RT congruency effects while standing. Yet, Smith et al. (2019) used color- and shape-discrimination tasks and found reduced (rather than increased) congruency effects and reduced switch costs (but only in error rates, not in RT) while standing relative to sitting. Hence, there is little consistency in the empirical findings concerning the influence of body balance on attentional control in task switching.

Using a different paradigm to study attentional control, Rosenbaum et al. (2017) examined the classic Stroop task (see MacLeod, 1991, for a review), in which participants had to name the letter color of a written color word. In this task, a processing conflict occurs when color and word meaning are incongruent relative to when they are congruent. These authors found that the Stroop effect (i.e., the color-word congruency effect) was smaller when standing than when sitting, and this effect has been replicated by Smith et al. (2019). However, recently, Caron et al. (2020) and Straub et al. (2022) could not replicate this effect in well-powered attempts at direct replication, and a meta-analysis does not reveal evidence for an effect of standing versus sitting on the Stroop effect, again showing inconsistency at the level of empirical findings (Straub et al., 2022).

At this stage we can summarize that even though balance does seem to have some effects on attentional control, the effects remain inconsistent and elusive. One reason for this inconsistency might be that these studies tested the cognition-motor (i.e., attention-balance) interaction without assessing balance control parameters themselves (as dependent variables). Instead, these studies took the sitting vs. standing comparison as an independent variable in terms of variation of balance control, which is a viable methodological approach for examining cognitive performance, but it cannot track potential mutual influences, such as an influence of cognitive control on balance control itself on a higher temporal resolution. Below we propose a method for examining effects of attentional control on balance control at finer detail but we first look into the existing research on this influence.

### Influence of cognitive control on balance control

Interference between cognitive tasks and the balance control during upright standing has been well documented. The typical methodological approach in early studies of the influence of attentional control on balance control employed dual-task requirements, in which motor performance either without or together with a cognitive task was assessed. In these studies, cognitive performance was tested in a sitting position as well. The combination of no change in balance performance^1^ and reduced performance (such as number of errors) in secondary working memory tasks (Brooks spatial and nonspatial memory tasks; visual spatial and object working memory) suggested that balance control involved domain-specific, spatial but not verbal cognitive resources (Kerr et al., 1985; VanderVelde et al., 2005). Note though that such studies typically used cognitive tasks of a more continuous nature, such as memory tasks (e.g., memory load tasks or backward counting), for which it is more difficult to examine cognitive–motor interactions at the level of specific subprocesses of tasks. In the present study, we focus on more typical attention tasks in which specific target stimuli required immediate speeded responses, so that we can take discrete stimulus–response (S–R) processing episodes as our basic unit of analysis as seen in tasks requiring response conflict resolution.

As argued earlier, in the present article we focus on cognitive attention tasks that consist of discrete S–R units for which it may be easier to analyze process-specific cognitive-motor interaction, and we focus on balance control during standing. For example, Melzer et al. (2001) used a modified Stroop task to impose a cognitive load during upright standing and reported that, relative to a control condition without cognitive task, body sway either decreased or increased in older adults depending on the stance width. With a narrow stance a decrease in sway was interpreted as increased stiffness due to muscular co-contractions in the leg. In contrast, dual-task standing with a wide stance increased body sway in both younger and older participants. Yet, body sway was assessed during periods of 20 s and a contrast between congruent and incongruent trials was not reported (Melzer et al., 2001). Similarly, Patterson et al. (2013) investigated the control of body sway while being engaged in a spatial Stroop-like conflict task. While standing upright, participants had to respond with an arm raise to the facing direction of an animated character presented on the left or right half of a projection screen. The condition with incongruent compared to congruent stimuli resulted in longer response times and reduced accuracy. In the conflict task overall, irrespective of the congruency condition, body sway was increased compared to a single-task stance condition. Again, however, any congruency effects at the individual trial level for the measures of body sway were not reported (Patterson et al., 2013).

Aggregate measures of balance control do not allow the examination of potentially subtle time-locked cognitive–motor interactions. Note, however, that there are also some good reasons why aggregate measures of balance control have been used in the past. For example, at a more systemic level, keeping body balance in quiet stance is an equilibrium process, which comprises oscillations of low frequency and has cycle durations of several seconds depending on the available sensory channels (Diener et al., 1982; Fitzpatrick et al., 1994). Aggregating over many trials decouples the time-variable cognitive processes preceding an observed motor response from the slower balance oscillations. In addition, given the mass of the body, there are also issues of biokinetic inertia, which can only be dealt with when taking longer periods of observation in order to calculate shifts in the center of pressure.

However, similar arguments are also true for neural measures of brain activity, such as in electrophysiology (e.g., EEG data) or with respect to the inertia of the hemodynamic response when measuring changes in regional blood flow (e.g., the BOLD response). In brain imaging, the first methodological approaches used blocked designs, but event-related approaches were soon developed (see D'Esposito et al., 1999) and these have some resemblance to measures of event-related potentials in EEG research (Heinze et al., 1990; Luck et al., 1990).

In summary, the study of the effect of performing attention-demanding tasks while standing has not resulted in clear conclusions with respect to the time course of processing in a given trial. We suspect that one reason for this is that the measurement of balance control is usually aggregated across a longer period of time (e.g., dozens of seconds or even in the range of minutes) in order to get more stable balance parameters as dependent variable so that the temporal resolution with respect to specific cognitive processes within an experimental trial is quite limited. Yet, from basic research on dual-task interference it is known that capacity sharing across tasks actually occurs at the micro-level of specific sub-processes during task execution, such as response selection (see Koch et al., 2018; Pashler, 1994, for reviews), and that scheduling processes to optimize dual-task performance can occur on a very fine temporal scale in the millisecond range. Hence, in order to study the interaction of attentional control and balance control it would be desirable to develop a research methodology that allows the researchers to examine the influence of cognitive processes on balance control in an “event-related” manner, within each experimental trial, with high temporal resolution.

### The present study

In the present study, we introduce an event-related approach to examine the influence of attentional control in a cognitive conflict task on balance control in upright standing at the temporal resolution of 150 ms time slices, which comes closer to the timing of individual cognitive processes within a given trial. To isolate cognitive processing conflict, we do not use the Stroop task, which, according to Kornblum et al.’s (1990) dimensional overlap model, includes a perceptual conflict and possibly a response conflict. Instead, we use the Simon task as an experimental paradigm that has often been taken to isolate cognitive conflict at the level of response selection (see Hommel, 2011 for review; Simon, 1969).

In the Simon task, participants are asked to respond with a left vs. right manual response to a non-spatial stimulus feature, such as stimulus shape. The critical variation pertains to the task-irrelevant stimulus location, with stimuli being presented either to the left or right of fixation. Numerous studies demonstrated that RTs are shorter if the required response and the irrelevant stimulus location are spatially congruent than if stimulus location and response side are spatially incongruent. The resulting congruency effect has been termed the Simon effect. In a recent review, Cespón et al. (2020) suggested that the task-irrelevant location of the target stimulus creates a spatial code that activates the spatially congruent response, which results in response facilitation on congruent trials, but incongruent trials lead to interference because the target location activates the incorrect response and thus creates a processing conflict with activating the instructed response. Here, we use the Simon task as an experimental tool to examine whether cognitive conflict at response selection would affect processes of balance control while standing. That is, we aim at assessing balance correlates of cognitive response conflict.

While there is consensus that the basic Simon effect is a measure of response-selection conflict (Hommel, 2011), there is less consensus about the interpretation of a specific empirical finding in the Simon task that refers to the sequential modulation of the congruency effect by the congruency level of the preceding trial (congruency sequence effect, CSE). The CSE represents the finding that the congruency effect is very robust following a congruent trial but reduced and sometimes even reversed after incongruent trials (Hommel et al., 2004; Stürmer et al., 2002). The CSE has been observed in other conflict tasks (e.g., in flanker compatibility task and Stroop task) as well and the specific underlying mechanisms are still under debate (see Braem et al., 2019; Frings et al., 2020, for recent discussions). The present study is not aimed at resolving this theoretical debate about the sequential modulation of the Simon effect (e.g., in terms of conflict monitoring vs. episodic binding, see Braem et al., 2019) but instead focusses on the influence of cognitive response conflict on balance control. Therefore, we restrict our analysis of the balance data on exactly those trials in which we could expect to find a robust Simon effect and thus on trials following congruent trials. For the Simon effect measured in these trials it is uncontroversial that it indexes response-selection conflict and the attentional processes required to resolve this conflict.

In the present study, we applied an event-related paradigm, in which the immediate effect of a single cognitive operation in the Simon task on body sway is determined from sway moment on a sub-second time scale. In order to distinguish between the effects of the time point of conflict and the time point of conflict resolution on balance control and in analogy to event-related potentials (ERPs) in EEG studies, we looked into the balance parameters of the force plate as a function of congruency in the Simon task in a target-aligned way, so that differences would reflect predominantly stimulus-based processing conflicts. Second, we looked into the balance parameters in a response-aligned way. Aligning data analysis to the response would isolate the effect of cognitive processes preceding the overt response production in the Simon task on balance control. We expected that if processes of cognitive conflict resolution and balance control interfered, then the condition with the strongest congruency effect should result in the maximum process overlap and effect sizes for the balance-related dependent variables.

Two different possibilities for the cognitive-balance interactions are studied in the present paper. First, we might predict that incongruent trials would lead to more variable sway due to less effective balance corrections. Increased variability of sway control could also result from specific response-related byproducts associated with finger presses in the more demanding incongruent condition. For example, anticipatory balance adjustments before a trigger button press could be modulated by target congruency. In contrast to the variability of sway as an expression of the balance control effort, we did not expect any deliberate or systematic alterations of the target state for balance control as expressed by the average force moment.

Second, and alternatively, cognitive conflict resolution could directly interfere with concurrent negative feedback control of balance, for example by the induction of cross-talk in conflict monitoring processes. Focussing on the temporal scale of balance-related (micro-) events, we might also assume that individual balance adjustments will be disrupted by conflict resolution, for example for the purpose of selecting an appropriate manual response to an ambiguous target. When processes of conflict resolution are engaged in the cognitive domain, then any processing bottlenecks might affect conflict resolution for the purpose of balance state estimation and correction. Thus, on a short timescale, the preparation and execution of required balance adjustments may be postponed or omitted, so that less sway variability could be observed. Finally, in order to assess if the general requirement to control standing balance alters performance in the cognitive task, participants were asked to perform the task in a sitting posture as well. Yet, this comparison is not relevant for the focus of this study and we only report the comparison of postures on the cognitive tasks for reasons of completeness.

## Methods

### Participants

Forty-eight healthy young adults (average age = 24.0 years, SD 4.1; female = 36, male = 12; right-handed = 44) were recruited for the current study. Participants were naïve regarding the hypotheses of the experiment, reported normal or corrected-to-normal vision and had neither neurological, musculoskeletal, psychiatric, nor any other relevant medical diagnoses and did not show any balance impairments. The sample size was chosen to provide a power of > 0.90 at an alpha of 0.05 for medium effect sizes (dz = 0.5) of simple comparisons using paired t-tests between congruent and incongruent trials. All participants were informed about the study protocol and gave signed written informed consent. The study protocol was conducted in accordance with the ethical research standards of the amended declaration of Helsinki.

### Materials and equipment

**Fig. 1:**
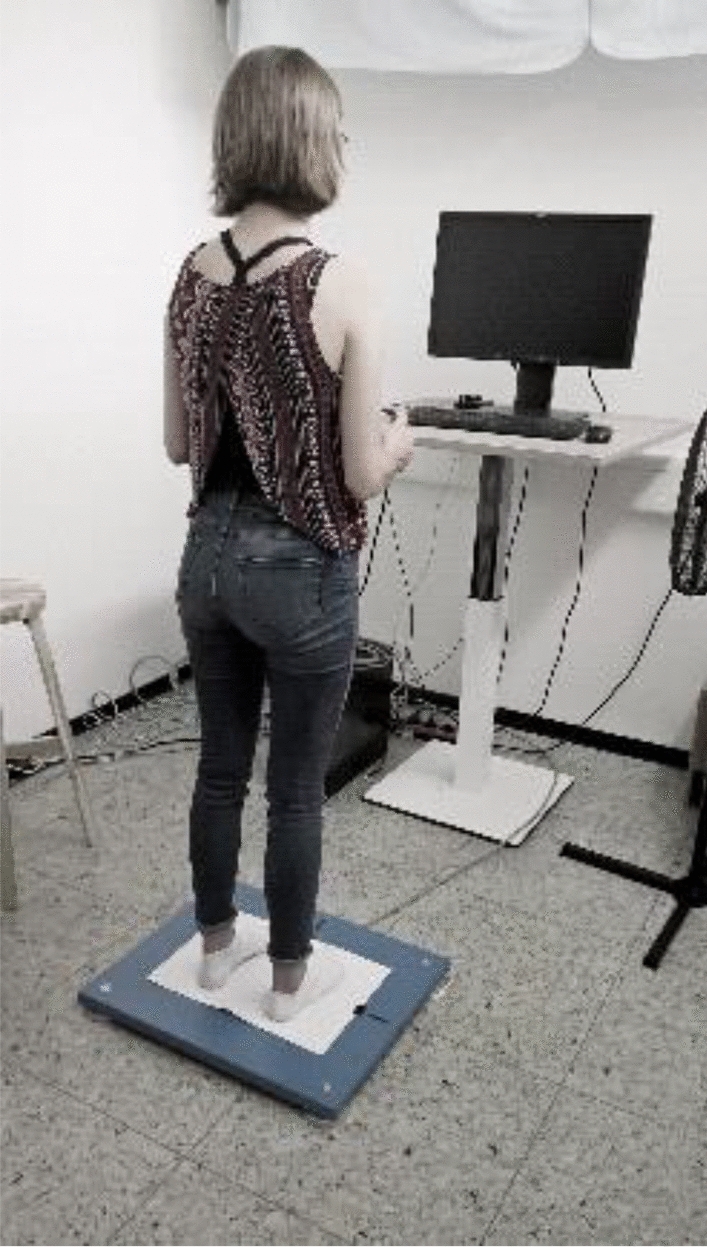
Experimental setup consisting of the force plate and the stimulus presentation display screen. The paper sheet on the force plate, on which the participant is standing, served for marking the foot prints to record and standardize each participant’s standing position. This allowed participants to step off the force plate during pauses in the experimental session

In a single session, participants were tested both in a seated position on a bar stool without back or armrests and in an upright standing posture. The order of standing or sitting was randomised for each participant, so that half of the participants started the lab session by performing the experimental task in sitting before standing, while the other participants started in a standing posture followed by sitting. No measures of balance control while sitting were obtained but manual reaction data only. Participants stood in normal lighting conditions with eyes open and with stockinged feet in quiet but relaxed normal bipedal stance with an intermalleolar distance of 21 cm centred on a portable force plate (Kistler Type 9260AA, Sindelfingen, Germany; see Fig. 1). The force plate data were sampled at a frequency of 1 kHz. A less challenging stance configuration was selected for reasons of ecological validity but also to reduce the likelihood that cognitive performance itself was negatively affected by the balancing demands.

Participants were instructed to keep both of their arms with flexed elbows close to their trunk so that the hands were level with the height of their navel. They did NOT receive instruction to stand as quietly as possible but only instructed to prevent any voluntary balance disturbance by moving body limbs or shifting weight. Participants held a manual trigger button in each hand and were asked to react as quickly as possible to a target stimulus, which was presented on a computer screen at the height of 145 cm. The distance between participant and screen was 113 cm. Size and width of the target stimuli on the screen was about 1 cm and their lateral position about 10.5 cm left or right of the fixation target. With an amplitude of 21 cm, the entire lateral viewing angle of the target stimuli was approximately 11 degrees.

### Procedure

Stimuli were presented electronically using the E-Prime 2.0 software (Psychology Software Tools, Pittsburgh, PA). The manual reaction task comprised a visual 2-alternative forced choice Simon task, in which the letters ‘T’ and ‘X’ were presented either on the left or the right of a central fixation. Fixation and target were presented simultaneously in white on a black background. The letter ‘T’ required participants to respond with the left trigger and the letter ‘X’ asked for a response with the right trigger. A match between the required manual response and the side of the target resulted in a congruent trial, while a mismatch between instructed target response and target location defined an incongruent trial.

At the start of each session, participants performed ten practice trials to familiarize themselves to the experimental procedure. Each participant performed ten blocks of 120 s duration of continuous standing. They were offered breaks between the individual blocks. Each of the ten blocks contained 80 trials so that 800 trials were presented in total. Following a fixation cross of 200 ms duration, a target was presented horizontally to the left or the right of the fixation target. The target remained visible until a manual response occurred or a period of 1.5 s had passed without response. The response latency period was followed by 350 ms feedback on the accuracy of the response and each trial ended with a 200 ms blank screen inter-trial-interval. Hence, the total response-target interval was 750 ms. For the data analysis, we considered only trials with RTs shorter than 1250 ms, that is, trials with overall durations shorter than 2 s. As RTs varied across trials, so varied the intertrial interval, too, so that slow oscillations in body sway should be decoupled from trial timing when aggregating over any individual trials (like in other event-related approaches).

### Post-processing of force plate data and parameter extraction

**Fig. 2:**
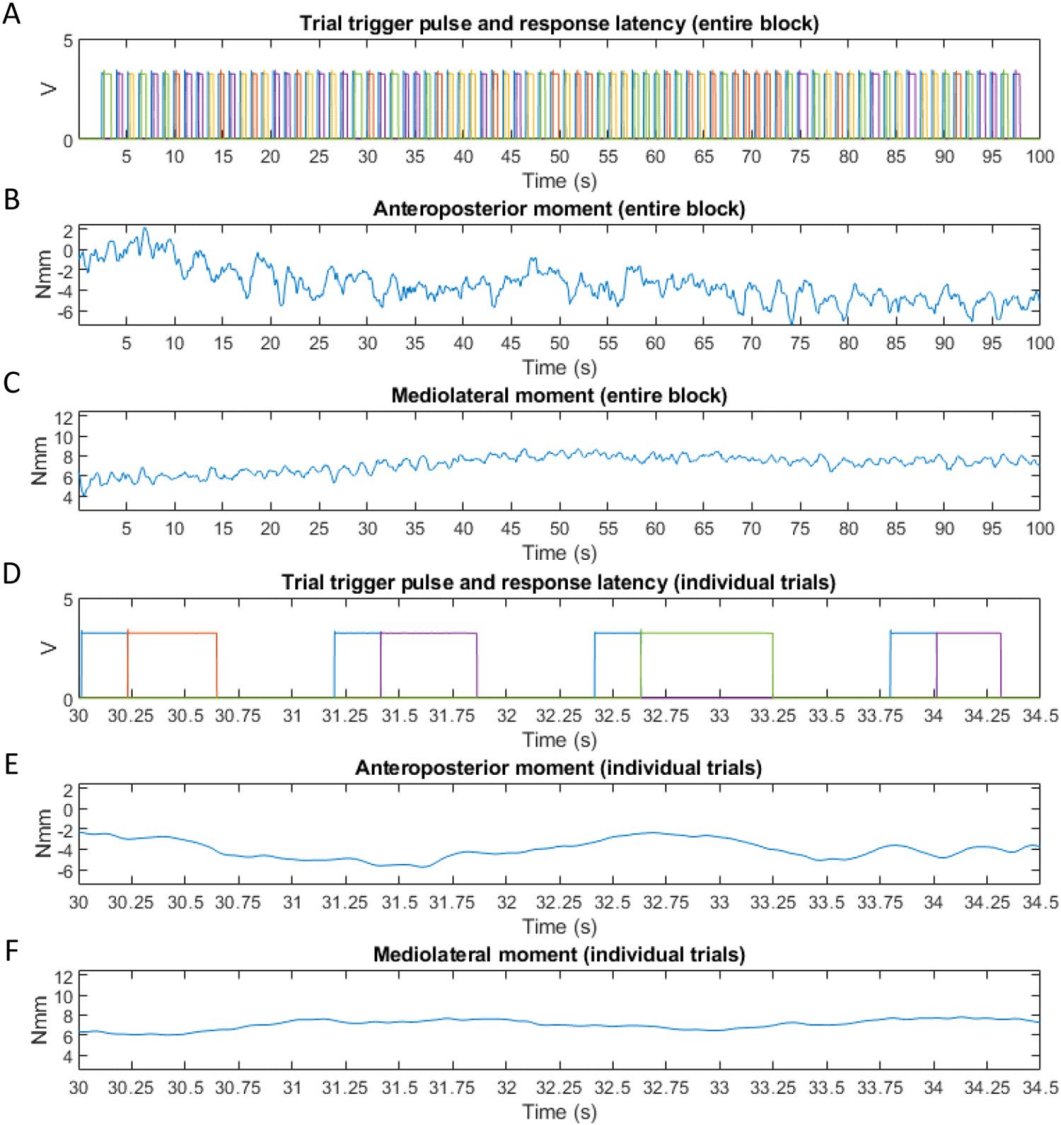
Illustrative event pulses and anteroposterior and mediolateral moments for an entire block (panels **A**–**C**) as well as for selected trial segments within this block (panels **D**–**F**) of an individual participant. Panels **A** and **D** (**D** is a temporal subset of **A**) show the period of the fixation cross (light blue colored trigger pulses) followed by the latency period of the subsequent manual response (red, green, purple, yellow colored pulses). Colored periods indicate response latencies encoding the specific target stimulus condition (congruent/incongruent; left/right). Panels **B** and **E** show the fluctuation of the anteroposterior moment, while the mediolateral moment is shown in the Panels **C** and **F** (color figure online)

Time series data acquisition was performed using BIOWARE datalogger software (Kistler Bioware 5.4.3.0 software). Trigger pulses provided by the experimental control software marking the phases of each trial were sent to the computer recording the force plate using a parallel port interface, where single pulse channels were registered as analog input devices sampled also at 1 kHz and synchronized with the force plate data acquisition (Fig. 2).

Time series data post-processing and parameter extraction were performed in MATLAB 2019b (The Mathworks, Natick, MA) using custom-prepared algorithms. Forces and moments of the force plate were used to characterize the control of body sway. A 4th order dual-pass Butterworth low-pass filter with 10 Hz cut-off frequency was applied to all time series data. Smoothing of the balance time series data is a common convention to remove high-frequency signals, artefacts or byproducts, which are not directly associated with supraspinal control of sway, such as electromagnetic noise or peripheral and spinal reactions (muscle twitches or spinal reflexes). In order to reduce local measurement noise, a 10 Hz cut-off (equivalent to an oscillatory signal’s shortest cycle duration of 100 ms) ensured that only supraspinal mechanisms of balance control were analysed such as long-latency (transcortical) reflexes or more complex balance-related behaviours (Christensen et al., 2000). In the following, testing blocks were segmented into individual trials using the event signals indicating the onset of fixation. Single trials were excluded from post-processing if the entire duration of a trial surpassed 2 s. These trials were labelled as “unresponsive” and were not considered for further processing. Additionally, trials with incorrect responses and correct trials following an incorrect response were excluded. In total, a proportion of 14% of all trials were excluded from data analysis, of which 5.9% (SD 4.9) were excluded as response errors, 5.2% as correct responses following an error, and 3.0% as correct responses with RTs longer than 1250 ms. Processed data and MATLAB scripts are available for download from the figshare.com database.

**Fig. 3:**
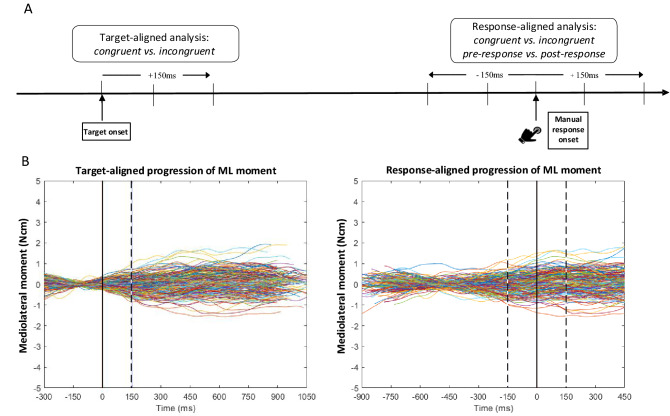
**A** A schematic representation of the extracted 150 ms time bins for target-alignment and response-alignment and the experimental factors. **B** Target-aligned (left panel) and response-aligned (right panel) illustrative fluctuations of the mediolateral moment of all trials for a single participant. The vertical black line indicates the timepoint of the target presentation (left panel) and manual reaction (right panel), respectively. Dashed vertical lines indicate the border of the time bins of 150 ms duration relative to the respective temporal reference used for statistical analysis. All trial segments were centered at the averaged value determined across the pre-target fixation period (fixation duration was 200 ms)

Two major branches of data processing were pursued by aligning all the time series data to the time points of the onset of the letter target (target-aligned) as well as when the manual response occurred (response-aligned; Fig. 3a). Around the anteroposterior (AP) and mediolateral (ML) axis for a specified time bin, the current state of balance was analysed as the absolute average (AV) ground reaction force moment, while the ongoing balance control effort was analysed in terms of the standard deviation (SD) of the ground reaction force moment. Variability of the force moment within a time bin expresses the amount of activity applied by the neuromuscular system for the control of standing balance. That is, greater variability would amount to balance adjustments being more likely to demonstrate their effect within this short period. In contrast, an average of the force moment represents the desired steady balance state during a specific time interval. Both measures, the average and the variability of the moment, represent complementary measures, such as the constant and variable performance error. Balance control parameters were computed across temporal bins of 150 ms duration.^2^ As a compromise between local temporal resolution and local noise reduction, a bin duration of 150 ms was chosen for integrating body sway control to assess interference with balance in the latency periods during which the target was processed (target-aligned), the response decision was made (first response-aligned bin), and the response was evaluated (second response-aligned bin). For the target-aligned sway data integration, one temporal bin beginning at the onset of the target was extracted, while for the response-aligned data, two bins were extracted—one before and one after the time point of the response. Spatial aggregates such as Centre-of-Pressure (CoP) position as well as the 1st or 2nd order derivatives thereof were considered redundant with respect to characterising balance control effort as CoP position is calculated from the moments and forces registered by the force plate and therefore were not chosen as outcome parameters.

### Design

Performance in the cognitive task, in terms of RT and error proportions, needed to be assessed to validate that a reliable effect of cognitive conflict was induced by the target stimuli. For the instructed manual reactions in the Simon task, the independent variables were congruency and previous-trial congruency. Moreover, for the sake of completeness, we included posture (i.e., sitting vs. standing) as an additional independent variable for the analyses of the performance data, even though our postural data come exclusively from the standing condition. Factors such as laterality of the target stimulus presentation and laterality of the manual reactions were not considered of interest due to lack of an a priori hypothesis because any specific effect of laterality would be cancelled out by calculating averages across sides.

The dependent variables (DVs) were response latency (RT) and error proportions. The DVs were based on response timing information acquired through the stimulus presentation software directly as well as by the event trigger signals sent to the data acquisition board of the force plate setup.

For the analysis of the force-plate data, we restricted our data analysis to trials following a congruent trial (i.e., only those trials in which we expected the strongest Simon effect). As dependent variables representing balance control in both the AP and ML directions of sway, we calculated the average and the standard deviation of the force moment time series within the respective time bins of each individual trial of a participant (Fig. 3b).

## Results

### Statistical analysis

All statistical computations were performed in R Studio 1.1.456.

For the force moment parameters, all statistical analyses were performed for the target-aligned and the response-aligned time series data. To explore which sway parameters might be sensitive to congruency, we analysed the average and standard deviation of the moments acting around either the anteroposterior and mediolateral axes within each 150 ms time bin. Paired *t* tests were calculated for a single bin following target onset in the target-aligned data and 2 × 2 ANOVAs with time bin and congruency as within-subject factors for response-aligned data. As we were interested in interpreting the mean of the mediolateral moment in relation to the hand that executed the manual response, the sign of the mean ML moment was recoded accordingly, so that a positive sign indicated a state directed towards the side of the manual response, while a negative sign indicated the opposite direction. Force moment parameters were natural log-transformed before statistical analysis to approximate a normal distribution. For the AV moment, however, the directional sign had to be dropped before natural log-transformation, so only absolute AV moment was processed. An alpha level of 0.01 was used to determine statistical significance. Descriptive statistics and inferential test statistics are summarized in supplementary tables (S1–S10).

### Analysis of manual reactions

#### Comparison between postures

**Fig. 4:**
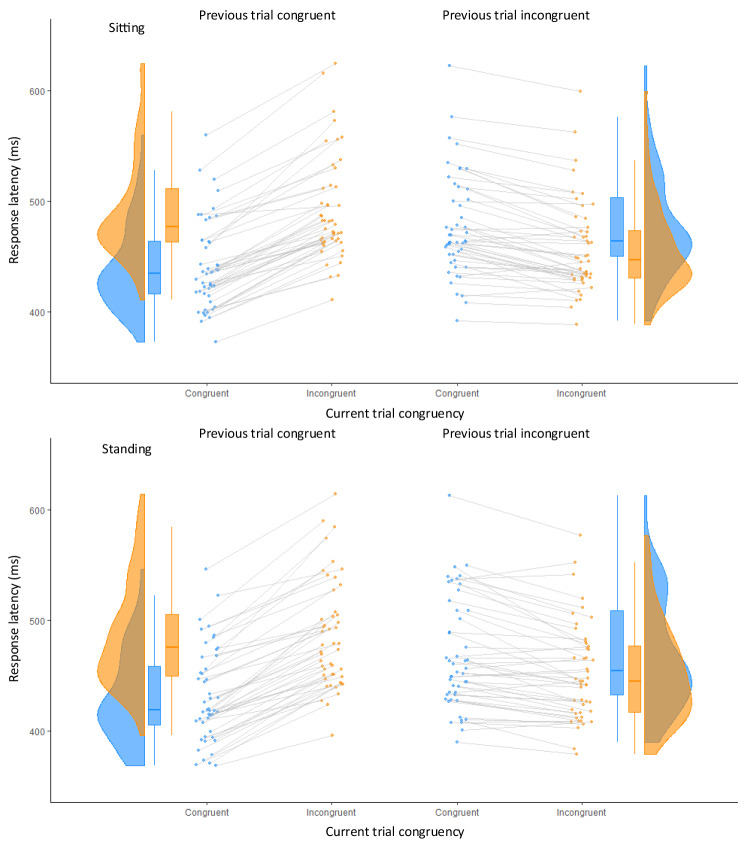
Raincloud plots for response latencies in the Simon task during sitting (top panels) and standing (bottom panels) as a function of current trial congruency and congruency of the previous trial. Panels on the left show performance when the previous trial was congruent, panels on the right when the previous trial was incongruent. Data in blue indicate performance in congruent trials, incongruent trials on orange (color figure online)

To look at effects of response selection conflict, RT and error proportions were analysed with congruency as within-subject factor and to assess specific sequential conflict adaptation effects, previous trial congruency was added as a second within-subject factor of the repeated-measures analyses of variance (ANOVAs). We additionally included posture (sitting vs. standing), even though the focus of the present study is on the postural data while standing. An ANOVA on RT (Table S3) across both postures (sitting and standing) indicated that RT tended to be shorter in standing (mean = 459 ms, SD 42) than sitting (mean = 466 ms, SD 41; *F*(1,47) = 5.22, *p* = 0.03, *partial eta*^*2*^ = 0.10). On congruent trials, RTs (mean = 454 ms, SD 42) were shorter than incongruent RTs (mean = 471 ms, SD 38; *F*(1, 47) = 95.25, *p* < 0.001, *partial eta*^*2*^ = 0.67). An interaction between previous trial congruency and congruency was found irrespective of the posture (*F*(1, 47) = 330.13, *p* < 0.001, *partial eta*^*2*^ = 0.88). When the previous trial was congruent, then RTs in congruent trials were shorter than in incongruent trials, resulting in a Simon effect of 52 ms, *t*(47) = 18.38, *p* < 0.001, *dz* = 2.68, but when the previous trial was incongruent, then RTs in congruent trials were even longer relative to RT on incongruent trials, resulting in a “reversed” Simon effect of 18 ms (*t*(47) = 7.56, *p* < 0.001, *dz* = 1.10; Fig. 4).

We also observed an interaction between posture and congruency (*F*(1, 47) = 7.39, *p* = 0.009, *partial eta*^*2*^ = 0.14), which indicated a minimally (but significantly) greater congruency effect in standing (mean = 18 ms, SD 32; *t*(47) = 9.54, *p* < 0.001, *dz* = 1.39) compared to sitting (mean = 15 ms, SD 32; *t*(47) = 8.62, *p* < 0.001, *dz* = 1.26). This interaction was independent of previous-trial congruency, (*F*(1,47) = 0.06, *p* = 0.80, *partial eta*^*2*^ < 0.01).

**Fig. 5:**
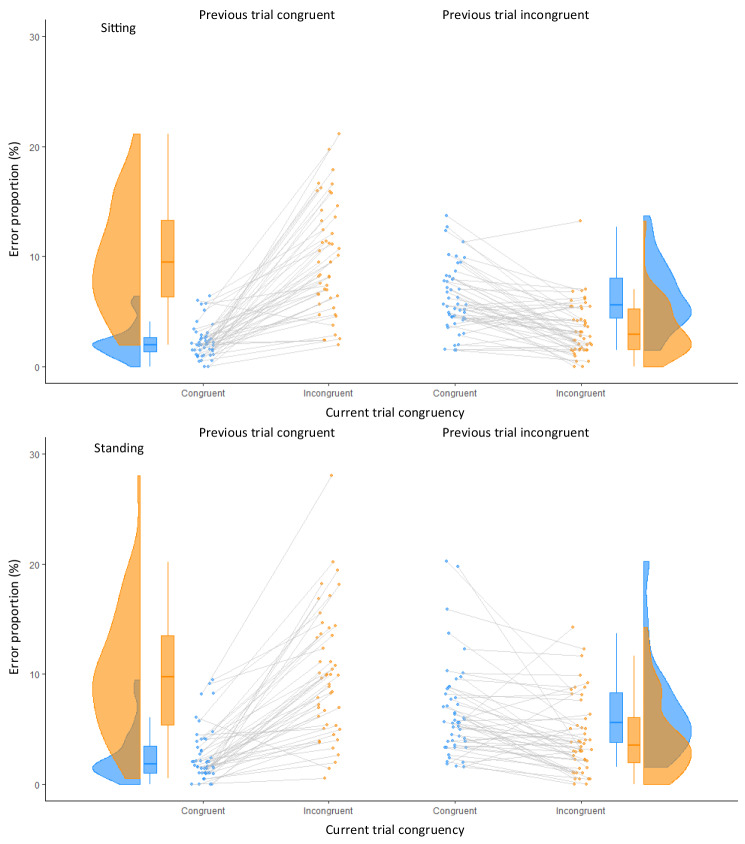
Raincloud plots for error proportions in the Simon task during sitting (top panels) and standing (bottom panels) as a function of current trial congruency and congruency of the previous trial. Panels on the left show performance when the previous trial was congruent, panels on the right when the previous trial was incongruent. Data in blue indicate performance in congruent trials, incongruent trials on orange (color figure online)

The error proportions demonstrated an effect of trial congruency (*F*(1, 47) = 34.16, *p* < 0.001, *partial eta*^*2*^ = 0.42) with more errors in incongruent trials (mean = 6.9%, SD 5.7) compared to congruent trials (mean = 4.4%, SD 4.4) and an effect of previous trial congruency (*F*(1, 47) = 22.73, *p* < 0.001, *partial eta*^*2*^ = 0.33) with higher error proportions (mean = 6.1%, SD 6.6) when the previous trial was congruent in contrast to when it was incongruent (mean = 5.2%, SD 3.7). An interaction between previous trial congruency and congruency was also found (*F*(1, 47) = 146.89, *p* < 0.001, *partial eta*^*2*^ = 0.76). When the previous trial was congruent, then error proportions in incongruent trials were higher than in congruent trials, resulting in a Simon effect of 7.4% (*t*(47) = 10.86, *p* < 0.001, *dz* = 1.58), but when the previous trial was incongruent, then error proportions in incongruent trials were even lower than in congruent trials, resulting in a “reversed” Simon effect of 2.5% (*t*(47) = 5.46, *p* < 0.001, *dz* = 0.80), just like in the RT data. A significant difference between the two postures did not occur (*F*(1, 47) = 3.17, *p* = 0.08, *partial eta*^*2*^ = 0.06; Fig. 5), and posture did not enter in any interaction (all *F*(1,47) < 1.14, all *p* > 0.29, all *partial eta*^*2*^ < 0.02).

#### Manual Simon effect of standing posture only

As the focus of our study was not on the comparison of sitting vs. standing but on the influence of response conflict on balance control while standing, we also analysed those data separately. The corresponding two-way ANOVA revealed shorter RTs on congruent trials than on incongruent trials, *F*(1,47) = 91.08, *p* < 0.001, *partial eta*^*2*^ = 0.66. The main effect of previous-trial congruency was not significant, *F*(1,47) = 3.59, *p* = 0.06, *partial eta*^*2*^ = 0.07, but, as expected, the interaction between previous trial congruency and congruency was significant *F*(1,47) = 230.94, *p* < 0.001, *partial eta*^*2*^ = 0.83. When the previous trial was congruent, then a Simon effect of 54 ms was observed, *t*(47) = 16.36, *p* < 0.001, *dz* = 2.39, but when the previous trial was incongruent, then a “reversed” Simon effect of 16 ms (incongruent trials resulted in shorter RTs than congruent trials) occurred, *t*(47) = 5.83, *p* < 0.001, *dz* = 0.85.

For the error rates, the ANOVA revealed a main effect of congruency, *F*(1,47) = 26.45, *p* < 0.001, *partial eta*^*2*^ = 0.36, showing higher error rates for incongruent trials than for congruent trials (7.1% vs. 4.6%) and thus a Simon effect of 2.5%. The main effect of previous-trial congruency, *F*(1,47) = 6.24, *p* = 0.02, *partial eta*^*2*^ = 0.12, tended to be significant. That is, when the previous trial was congruent, error proportions were higher (6.3%) than when the previous trial was incongruent (5.5%), which expresses generally improved performance after experiencing response selection conflict. Importantly though, the interaction was significant, *F*(1,47) = 111.39, *p* < 0.001, *partial eta*^*2*^ = 0.70. When the previous trial was congruent, then error proportions in congruent trials were lower than in incongruent trials, resulting in a Simon effect of 7.3%, *t*(47) = 10.07, *p* < 0.001, *dz* = 1.47, but when the previous trial was incongruent, then, consistent with the RT data, error proportions on congruent trials were even higher than on incongruent trials, resulting in a “reversed” Simon effect of 2.2%, *t*(47) = 3.69, *p* < 0.001, *dz* = 0.54.

In sum, we found a very pronounced congruency sequence effect. As we are not interested in the congruency sequence effect itself, we restricted the data analyses of the force-plate data to the trials following congruent trials. These are the trials in which we can expect to measure balance correlates of manual response conflict.

### Analysis of balance parameters from the force-plate data

#### General sway modulation across a cognitive trial

**Fig. 6:**
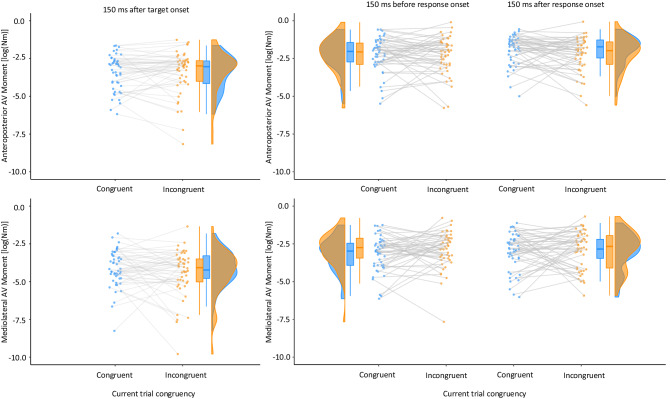
Raincloud plots for the log-transformed, absolute average (AV) moment in the anteroposterior direction (top panels) and the mediolateral direction (bottom panels) as a function of congruency of the current trial (and for trials where the previous trial was congruent only) for each extracted temporal bin (left panels: across 150 ms after target onset; middle panels: across 150 ms before response onset; right panels: across 150 ms after response onset). Data in blue indicate performance in congruent trials, incongruent trials on orange (color figure online)

**Fig. 7:**
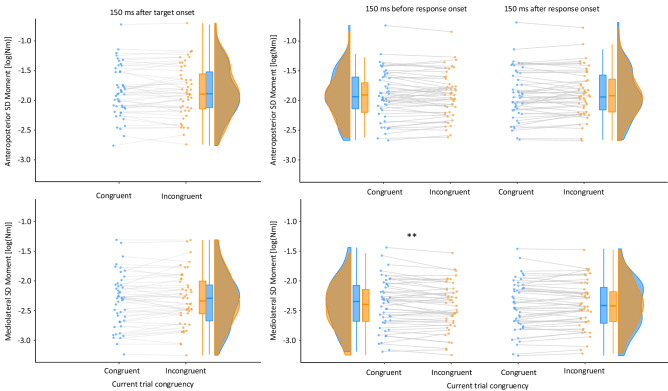
Raincloud plots for the log-transformed standard deviation (SD) of the moment in the anteroposterior direction (top panels) and the mediolateral directions (bottom panels) as a function of congruency of the current trial (only for trials where the previous trial was congruent) for each extracted time bin (left panels: across 150 ms after target onset; middle panels: across 150 ms before response onset; across right panels: 150 ms after response onset). Data in blue indicate performance in congruent trials, incongruent trials on orange. Double-asterisks indicate *p* < 0.01

Focusing on the condition with upright standing, irrespective of trial congruency, sway was modulated across a typical trial with changes from the post-target and the pre- and post-response periods (Table S8). The absolute average force moment differed as a function of time bin (*F*(2,94) = 44.01, *p* < 0.001, *partial eta*^*2*^ = 0.48) in the AP direction (Fig. 6). Paired *t* tests between time bins showed that the average moment deviated less in the 150 ms after target onset compared to both the 150 ms before and after response onset (both *t*(47) > 7.77, both *p* < 0.001, both *dz* > 0.80). No difference was observed between the time bins before and after response onset (*t*(47) = − 1.56, *p* = 0.12, *dz* = 0.16). In the ML direction, an effect of time bin was also found (*F*(2,94) = 54.16, *p* < 0.001, *partial eta*^*2*^ = 0.54), which indicated a lateral modulation of the average moment across the progression of a trial. Again, the deviation was less following the target onset compared to both time bins before and after response onset (both *t*(47) > 7.90, both *p* < 0.001, both *dz* > 0.81); Fig. 7, both rows.

Also the variability of the force moment also showed a modulation across the time bins of an entire trial in both the AP (*F*(2,94) = 8.86, *p* < 0.001, *partial eta*^*2*^ = 0.16) and the ML (*F*(2,94) = 41.26, *p* < 0.001, *partial eta*^*2*^ = 0.47) directions (Fig. 7, both rows). Paired *t* tests between time bins showed that, relative to the variability during the 150 ms following the onset of the target, the AP and the ML moments were less variable both in the 150 ms before (AP: *t*(47) = 4.31, *p* < 0.001, *dz* = 0.44; ML: *t*(47) = 8.47, *p* < 0.001, *dz* = 0.87) and after (AP: *t*(47) = 3.14, *p* = 0.002, *dz* = 0.32; ML: *t*(47) = 7.49, *p* < 0.001, *dz* = 0.77) the response onset. Numerically, the 150 ms before response onset differed from after response onset in the AP direction (*t*(47) = 1.79, *p* = 0.08, *dz* = 0.18) but not in the ML direction (*t*(47) = 0.40, *p* = 0.69, *dz* = 0.04). Finally, an interaction between time bin and trial congruency was observed (*F*(2,94) = 6.55, *p* = 0.002, *partial eta*^*2*^ = 0.12), which will be analysed in detail in the sections below.

#### Target-aligned time series analysis of local congruency effects.

We analysed these data using paired *t* tests with congruency as independent variable. The analyses of the moment of the ground-reaction force within the 150 ms temporal bin following the onset of the target did not result in significant effects of congruency neither for the average nor for the variability of the moment in neither the ML nor the AP direction, all *ps* > 0.22, all *dz*s < 0.18 (Figs. 6 and 7, left panels; Table S10).

#### Response-aligned time series analysis of local congruency effects

Next, we analysed the time series data for all balance control parameters aligned by the onset of the manual response. To this end, we calculated means aggregated over 150 ms bins before and after the response onset, so that the ANOVA included congruency and time bin as independent variables (Table S10). Across the two time bins, both the average absolute AP and ML moments were not affected by time bin or current trial congruency and did not show an interaction between both factors (all *F*(1,47) < 1.91, all *p* > 0.17., all *partial eta*^*2*^ < 0.04).

The variability of the AP moment did not show an effect of congruency or a change across the time bins but the numerical tendency of an interaction between time bin and congruency (F(1,47) = 3.02, p = 0.09, *partial eta*^*2*^ = 0.06). (Fig. 7 upper middle and upper right panels). However, paired t-tests showed no effect of congruency in either the 150 ms time bins before or after response onset, both *p* > 0.25. In contrast, the variability of the ML moment showed an interaction between time bin and congruency, *F*(1,47) = 8.87, *p* = 0.005, *partial eta*^*2*^ = 0.16 (Fig. 7 lower middle and lower right panels). Paired t-tests showed that in the 150 ms time bin before onset of the manual response, incongruent trials showed reduced variability of the ML moment, *t*(47) = 2.69, *p* = 0.009, *dz* = 0.39, while no effect of congruency was present in the 150 ms after response onset, *t*(47) = 0.66, *p* = 0.51, *dz* = 0.10.

## Discussion

In the present study, we assessed measures of balance control while participants were standing on a force plate in order to develop an event-related approach sensitive to detect interference between balance control and control of cognitive conflict in the range of sub-second time periods. Specifically, we had participants perform a Simon task, which requires resolution of conflict during selection of left vs. right manual button-press responses. We showed that spatial response conflict in the cognitive Simon task may have affected balance control in terms of a greater reduction in mediolateral body sway variability within a short period preceding the execution of the instructed manual reactions. In contrast, there was neither such cognitive-motor interference associated with target encoding in the Simon task nor with or after response execution. These observations may indicate that process-specific effects in a cognitive task related to response selection carry over to the balance domain.

### Performance in the Simon task: effects of posture and of congruency sequence

Overall, we observed a congruency effect of 17 ms (i.e., the Simon effect). When including the posture (sitting vs. standing), we found that there was a small but significant increase of the congruency effect during standing compared to sitting (18 vs. 15 ms). This finding is in line with the notion that the greater complexity of standing caused interference and performance reduction in cognitive control. This occurred despite our intention to assess participants’ performance in a comparatively easy standing posture. It is notable that this finding is not in line with recent observations of reduced congruency effects while standing (Rosenbaum et al., 2017) and more in line with previous findings questioning a reduction of congruency effects in a standing posture (Caron et al., 2020; Straub et al., 2022). However, the focus of our present study was on the effect of congruency on balance control during standing.

For our purpose of focusing on the influence of response conflict on balance control (see below), it is important to note that we also found a massive congruency sequence effect in performance. The congruency effect was 52 ms following a congruent trial but was actually reversed following an incongruent trial, representing the predicted and well-established congruency sequence effect (see Egner, 2007, for a review). In order to avoid any interactions between the congruency effects of the current and previous trials, we only analysed trials that followed a congruent previous trial because these represent the trials in which we could expect a reliable Simon effect reflecting the influence of response conflict on response selection.

### Analysis of balance control using body sway parameters

While we hypothesized that resolution of response selection conflict would interfere with the control of body sway based on a wealth of previous literature on such interactions assessed by aggregating across much longer time periods, it was important to show that our effects would be related to the occurrence of specifically defined cognitive processes. Moreover, while we could predict that the cognitive process of response selection is critical, based on neurocognitive findings (see Cespón et al., 2020, for a review), there was no prior knowledge of which aspect of body sway control would be susceptible and sensitive to any cognitive interference. Therefore, we analysed the average moment in each direction as an expression of balance-related state, and we analysed the variability of the moments as an indication of the balance control effort imposed on body sway.

Both body sway parameters (AV and SD moment) expressed a processing stage-dependent modulation across the time course of an entire trial. In the 150 ms after target onset, that is in the stimulus processing stage, the average force moment deviated less from zero in both directions of sway in comparison to the response selection and response execution stages. As Fig. 3 illustrates for an individual participant, this is to be expected due to the centring of the force moments relative to the average determined across a trial’s pre-target fixation period. From presentation of the target onwards, the force moment will increase its distance from the zeroed state until it settles at the time of the response. However, the congruency condition of a particular trial did not have any effect on this dynamical indicator. These observations merely validate the sensitivity of the methodological approach.

More relevant to the research questions posed in this study is that the variability within time bins demonstrated a reduction by about 8% from the period after target presentation towards execution of the response. This indicates that response execution (manual trigger press) in itself did not cause any physical movement artefacts. While target presentation resulted in greater local variability than response execution, we found that cognitive conflict during spatial left vs. right responses reduced variability of the mediolateral moment in the 150 ms time bin before response onset in incongruent trials further, that is, when response selection conflict was resolved and/or inaccurate response tendencies were suppressed. In this situation a mediolateral variability reduction of approximately 14% occurred. Hence, using a new event-related methodology to force plate data, we found a specific balance correlate for cognitive spatial response conflict that did not generalize to anterior–posterior movements or to time bins for which such effects were not predicted beforehand (i.e., with target encoding or only after response execution).

Note that our finding of reduced sway variability in ML moment in incongruent trials relative to congruent trials is specific in many respects. (i) Different from previous studies that examined the control of body sway in traditional measures with low temporal resolution, we investigated body sway variability on a very short timescale, (ii) the congruency effect is only visible when the moment time series data were aligned by the onset of the manual reactions, (iii) the influence of trial congruency was limited to the mediolateral direction of sway during the selection phase of the correct manual response, the plane in which the directional conflict exists, and (iv) only the temporal bin integrating the 150-ms period before the manual response was susceptible to the congruency condition of the target. In contrast, the target-aligned 150 ms time bin following target presentation showed no effect of congruency condition. Together, the data strongly suggest that in the bin 150 ms prior to response onset, processes related to response selection dominate activity instead of processes related to target processing.

We designed our analytical approach after event-related neurophysiological studies of the Simon task (see Cespón et al., 2020, for a review). The relatively high sample rate of 1 kHz that we employed to acquire body sway responses as well as the alignment of the traces of the ground reaction force moments by either the onset of the visual target or the onset of the manual response enabled us to look for spatiotemporal commonalities in the time series data. In contrast to Simon task effects expressed by altered event-related potentials, however, we did not find a specific Simon effect on the average moment before or after the response. A reason may be that the fluctuation of the moment does not seem to possess a characteristic or stereotypical waveform that could allow time series superposition for the detection of specific spatiotemporal features as exemplified in Fig. 3. Note though that this impression does not preclude the possibility that, in the future, more sensitive data analytical procedures could potentially identify fluctuations in the average moment that distinguish between conditions with and without conflict during selection of the manual reaction.

The reduction in the mediolateral variability of the moment of the ground reaction force in incongruent trials may be an expression of a lowered probability for a direction-specific balance adjustment to occur during the cognitive selection of a manual response if there is a response conflict to be resolved. As the time point of presentation of a target stimulus was quasi-jittered in relation to the time point at which a balance correction had to be performed by the randomization of a trial’s congruency condition and by the variability of a participant’s past response latencies, we assume that interference between the requirement to adjust balance and a conflict in response selection did not emerge in every single incongruent trial but in the statistical majority of those trials. Nevertheless, future studies might choose to add deliberate jitter to the inter-trial-intervals to further decouple the regularity of stimulus presentation from regularities in balance control.

It is possible that cross-talk between the cognitive task and balance control was caused either by body movements associated with the manual reactions or by target-oriented saccades or gaze shifts. The preparation or execution of either actions might have had a direct impact on body sway. However, these effects ought to have affected trials with congruent and incongruent targets to the same degree. Also, target position and any associated saccades and bodily orienting responses were uncorrelated with the congruency variable as target location was not the response-relevant feature.

In order to explain the differences in response-locked sway control variability between congruent and incongruent trials, one would have to assume differences in the characteristics of these motion and oculomotor byproducts. Future studies ought to record pressure forces at the trigger button, hand and body kinematics and eye movements to control for these possible confounds. An argument against possible influences of response button presses on body sway is the observation that variability of sway is numerically greater during the target perceptual stage than the response stage. A manual pressure-related byproduct should evoke increased variability on the latter stage.

From the dual-task literature it is well known that two capacity-limited processes interfere with each other (i.e., produce a processing bottleneck) when required on a very short time scale of only dozens of milliseconds, and this interference has been termed “psychological refractory period” (PRP) effect (for reviews see Koch et al., 2018; Pashler, 1994). In the present experimental conditions with presumably high response conflict in incongruent trials, we believe that conflict-induced delay of response selection in the Simon task can propagate to regularly triggered, small balance corrective adjustments. Overlap between capacity-limited “central” cognitive processes, required for resolution of response conflict and the effortful selection of a manual response, and processes involved in the triggering of a balance correction may have created a transient “micro-bottleneck”. More specifically, these coincidental micro-bottlenecks could occur because balance corrections that would have been triggered close in time to the independently triggered response selection process in the Simon task may have been altered or omitted because of an (i) absence, (ii) a partial reduction or (iii) a delay in an intermittent balance control signal. For example, Loram and colleagues (2011) assumed that feedback control of body balance is a serial, ballistic process in which the balance state is observed continuously but adjustments occur in an intermittent, predictive open-loop fashion (Gawthrop et al., 2011; Loram et al., 2011). While peripheral mechanisms for balance control are supposed to have a high processing bandwidth, the bandwidth of central balance control is considered relatively low. For example, relatively long feedback time delays of latencies longer than 150 ms indicate low bandwidth but more flexible control of the direction and amplitude of body sway by the involvement of intentional control mechanisms in a context-specific manner (Loram et al., 2009). Thus, as our Simon task may have created conflict in spatial terms along the mediolateral (body) axis, the equivalence with a congruency effect on mediolateral balance control is a plausible finding. Possibly the contextual demands of the Simon task implicitly imposed direction-specific constraints on the balance control of ML sway. For example, the Simon task demands may have interfered more selectively with neuromuscular control of ML sway via the hips compared to AP sway controlled by the ankles (Winter et al., 1996).

Processes of cognitive conflict resolution may play a direct role in balance control such as for the resolution of intersensory conflict (Redfern et al., 2009). Recently, Redfern and colleagues (2018) observed relationships between body sway in conditions with differing demands on intersensory conflict resolution and diverse cognitive functions, such as decision speed, control of cognitive conflict and abilities of visuospatial processing and memory. Performance in a cognitive conflict task correlated with sway especially when intersensory conflict was induced by sway-referenced visual feedback and a fixed support base (Redfern et al., 2018). The authors concluded that visual cognitive conflict resolution shares processes with sensory integration and intersensory conflict resolution when balance control relies predominantly on somatosensory afferences and a relative down-weighting of vision.

An important distinction between theoretical approaches that assume an intermittent balance control scheme is the question whether any balance corrections are performed at regular temporal intervals or in an event-dependent fashion, by which a balance adjustment is triggered when the estimated balance state, either measured or predicted, transgresses a threshold criterion. The decision at which point to activate and deactivate specific muscles could be made by a high-level monitoring process that considers the rate of change in the stability component in a phase space representation (Tanabe et al., 2017). Thus, it seems justified to conclude that balance control incorporates high- and low-level processes and may be intermittent. While low-level and more automatic processes may be regulating the maintenance of postural stiffness and damping at a higher temporal frequency, high-level processes monitoring and predicting states of balance stability may show periods of “cognitive neglect” lasting 150 ms and longer when engaged in cognitive response conflict resolution.

We did not use secondary-task methodology to analyze cognitive-motor interference but instead an event-related approach examining interference at the level of specific processes with high temporal resolution. Previous observations using the traditional secondary-task methodology, where measures of cognitive and balance performance were contrasted across blocked conditions, reported sway reduction as the result of an adopted balance control strategy (Brown et al., 2002). The assumption that the balance control system strategically increases postural stiffness, however, does not apply to the methodology used in our present study. Here congruent and incongruent trials alternated randomly in short intervals and which, therefore, rendered rapid switching between balance control strategies following target presentation implausible. Instead, it is more reasonable to assume that during the entire experiment, an individual’s mode of balance control rested in a single default state of sensorimotor organization.

A limitation of the present study is its exploratory nature so that a replication in the same or in similar experimental paradigms would seem desirable. In fact, the present paper suggests that cognitive-balance interference can be uncovered using an event-related approach. Still, the observed selectivity of the reported findings might be a spurious result, effectively leading to a false-positive erroneous conclusion. The question at this point is, however, what can be gained from the assumption that cognitive control does interfere with the control of balance on the global level of task blocks, as seen in studies that pursued the traditional multitasking approach, but NOT on the level of individual cognitive trials? The assumption that conflict resolution has no effect on balance control at a local level or only when it occurs as a uniform effect across all trial phases, both directions of sway and in all complementary performance parameters, is of a very strong order. This assumption would in its nature disregard many aspects of our current understanding about the selectivity of cognitive control processes as well as the known sensitivity of balance control to the context of a specific task. Nevertheless, the experimental methodology for investigating the interaction between balance and cognitive control ought to be further extended and improved in order to detect the omission or delay of single balance adjustments.

### Conclusions

In the present study, we used a visuospatial Simon task to examine cognitive–motor interaction in measures of balance control. Specifically, we developed an event-related approach and demonstrated, with very high temporal resolution, an alteration of short-term body sway control during cognitive processing and response selection. Thus, we provided first evidence that cognitive conflict resolution may interfere with the effort to control body sway, which we observed in terms of reduced mediolateral control variability. As this reduction occurred in response-aligned sway time series data and in the 150 ms before the onset of the manual response, which coincides with the response selection stage, we propose the presence of a specific micro-bottleneck. The sharing of a common processing capacity in this period involved in the postponement or suppression of an incongruent response tendency may structurally disturb the monitoring and the adjustment of the current balance state.

## Supplementary Information

Below is the link to the electronic supplementary material.

Supplementary file1 (DOCX 30 KB)

## Data Availability

The data generated and analysed during this study are available in the Figshare repository, 10.6084/m9.figshare.22190155.

## Footnotes

1 Here no change in balance performance means CoP steadiness in tandem Romberg stance, where feet are placed in a heel-to-toe position, with one foot directly in front of the other, in terms of the average absolute distance from mean, the standard deviation of absolute distance from mean, average mediolateral distance from mediolateral mean, the average anterior–posterior distance from anterior–posterior mean, the mediolateral range, and the anteroposterior range.

2 As shortest response latencies rarely fell below 200 ms, a period of 150 ms ensured that (i) processes associated with manual response execution were not considered for target-aligned analysis and (ii) processes associated with visual target encoding were not considered for response-aligned analysis.
