# Supplementary material for: Assessing the influence of cognitive response conflict on balance control: an event-related approach using response-aligned force-plate time series data
Source: Psychol Res. 2023 Mar 2;87(7):2297–315. doi: 10.1007/s00426-023-01809-9 (PMC10457244; doi:10.1007/s00426-023-01809-9)
Supplement: Supplementary file 1 — Supplementary file1 (DOCX 30 KB) [file 426_2023_1809_MOESM1_ESM.docx]

Running head: COGNITIVE-POSTURAL INTERFERENCE IN THE SIMON TASK

Assessing the influence of cognitive response conflict on balance control – An event-related approach using response-aligned force-plate time series data

Leif Johannsen^1^, Denise Nadine Stephan^1^, Elisa Straub^2^, Falko Döhring^3^, Andrea Kiesel^2^, Iring Koch^1^, & Hermann Müller^3^

^1^ Institute of Psychology, RWTH Aachen University, Germany

^2^ Department of Psychology, University of Freiburg, Germany

^3^ Department of Sport Science, University of Gießen, Germany

Supplementary tables.

Table S1.

Descriptive statistics of the manual reaction latencies by posture, current and previous trial congruency

| Posture | Current trial  congruency | Previous trial  congruency | Average (ms) | Confidence  interval (ms) |
| --- | --- | --- | --- | --- |
| Sitting | congruent | Congruent | 440.76 | 4.95 |
| Sitting | Congruent | Incongruent | 476.68 | 4.95 |
| Sitting | incongruent | Congruent | 490.65 | 4.68 |
| Sitting | Incongruent | Incongruent | 457.30 | 4.28 |
| Standing | Congruent | Congruent | 431.35 | 4.89 |
| Standing | Congruent | Incongruent | 468.37 | 5.34 |
| Standing | Incongruent | Congruent | 485.40 | 4.87 |
| Standing | incongruent | Incongruent | 452.26 | 4.20 |

Table S2.

Descriptive statistics of the manual error proportions by posture, current and previous trial congruency

| Posture | Current trial  congruency | Previous trial  congruency | Average (%) | Confidence  interval (%) |
| --- | --- | --- | --- | --- |
| Sitting | congruent | Congruent | 2.26 | 0.79 |
| Sitting | Congruent | Incongruent | 6.26 | 0.57 |
| Sitting | incongruent | Congruent | 9.76 | 0.97 |
| Sitting | Incongruent | Incongruent | 3.40 | 0.66 |
| Standing | Congruent | Congruent | 2.62 | 0.61 |
| Standing | Congruent | Incongruent | 6.60 | 0.93 |
| Standing | Incongruent | Congruent | 9.90 | 1.11 |
| Standing | incongruent | Incongruent | 4.37 | 0.58 |

Table S3.

Test statistics for ANOVAs of manual reaction latencies and error proportions with (rows 1 and 2) posture as within-subject factor and without (standing only; rows 3 and 4). Significant effects or interactions are indicated with asterisks.

|  | F, p, partial eta^2 | | | | | | | |
| --- | --- | --- | --- | --- | --- | --- | --- | --- |
|  | Main effect | Main effect | Main effect | Interaction | Interaction | Interaction | Interaction |  |
|  | Posture | Current trial  congruency | Previous trial  Congruency | Posture by  current trial  congruency | Posture by  previous trial  congruency | Current trial  congruency by  previous trial  congruency | Posture by  current trial  by previous  trial congruency |  |
| Reaction latencies | 5.22,  .03, .10 | 95.25,  <.001, .67 *** | 4.53,  .04, .09 | 7.39,  .009, .14 ** | 0.25,  .62 | 330.13,  <.001, .88 *** | 0.06,  .80 |  |
| Error proportion | 3.17,  .08, .06 | 34.16,  <.001, .42 *** | 22.73,  <.001, .33 *** | 0.41,  .53 | 0.94,  .34 | 146.89,  <.001, .76 *** | 1.14,  .29 |  |
| Reaction latencies |  | 91.08,  <.001, .66 *** | 3.59,  .06, .07 |  |  | 230.94,  <.001, .83 *** |  |  |
| Error  proportions |  | 26.45,  <.001, .36 *** | 6.24,  .02, .12 |  |  | 111.39,  <.001, .70 *** |  |  |

Table S4.

Descriptive statistics of the target-aligned time series data (absolute AV Moment) by direction of body sway (AP: anteroposterior; ML: mediolateral) and current trial congruency

| Direction | Time bin | Current trial congruency | Average (Nm) | Confidence  interval (Nm) |
| --- | --- | --- | --- | --- |
| AP | 150 | Congruent | 0.055 | 0.008 |
| AP | 150 | Incongruent | 0.062 | 0.011 |
| ML | 150 | Congruent | 0.028 | 0.007 |
| ML | 150 | Incongruent | 0.027 | 0.009 |

Table S5.

Descriptive statistics of the target-aligned time series data (SD Moment) by direction of body sway (AP: anteroposterior; ML: mediolateral) and current trial congruency

| Direction | Time bin | Current trial congruency | Average (Nm) | Confidence  interval (Nm) |
| --- | --- | --- | --- | --- |
| AP | 150 | Congruent | 0.171 | 0.004 |
| AP | 150 | Incongruent | 0.173 | 0.004 |
| ML | 150 | Congruent | 0.107 | 0.003 |
| ML | 150 | Incongruent | 0.110 | 0.003 |

Table S6.

Descriptive statistics of the response-aligned time series data (absolute AV Moment) by direction of body sway (AP: anteroposterior; ML: mediolateral), time bin, and current trial congruency

| Direction | Time bin | Current trial  congruency | Average (Nm) | Confidence  interval (Nm) |
| --- | --- | --- | --- | --- |
| AP | -150 | Congruent | 0.168 | 0.039 |
| AP | -150 | Incongruent | 0.173 | 0.036 |
| AP | 150 | Congruent | 0.198 | 0.035 |
| AP | 150 | Incongruent | 0.172 | 0.037 |
| ML | -150 | Congruent | 0.071 | 0.020 |
| ML | -150 | Incongruent | 0.092 | 0.020 |
| ML | 150 | Congruent | 0.084 | 0.020 |
| ML | 150 | Incongruent | 0.099 | 0.021 |

Table S7.

Descriptive statistics of the response-aligned time series data (SD Moment) by direction of body sway (AP: anteroposterior; ML: mediolateral), time bin, and current trial congruency

| Direction | Time bin | Current trial  congruency | Average (Nm) | Confidence  interval (Nm) |
| --- | --- | --- | --- | --- |
| AP | -150 | Congruent | 0.162 | 0.004 |
| AP | -150 | Incongruent | 0.159 | 0.004 |
| AP | 150 | Congruent | 0.164 | 0.004 |
| AP | 150 | Incongruent | 0.165 | 0.004 |
| ML | -150 | Congruent | 0.100 | 0.002 |
| ML | -150 | Incongruent | 0.095 | 0.002 |
| ML | 150 | Congruent | 0.098 | 0.002 |
| ML | 150 | Incongruent | 0.098 | 0.002 |

Table S8.

Test statistics for ANOVAs of the time course across an entire trial by combining target-aligned and response-aligned, log-transformed average (unsigned) and standard deviation of force moments by direction of body sway (AP: anteroposterior; ML: mediolateral), time bin, and the current trial congruency. Significant effects or interactions are indicated with asterisks.

| Moment |  | F, p, partial eta^2 | | | |
| --- | --- | --- | --- | --- | --- |
| Parameter | Direction | Main effect | Main effect | Interaction |  |
|  |  | Time bin | Current trial  congruency | Time bin by  current trial  congruency |  |
| AV | AP | 44.01,  .001, .48 *** | 0.54,  46, .01 | 1.23,  .30, .03 |  |
| AV | ML | 54.16,  < .001, .54 *** | 0.06,  .80, < .01 | 1.95,  .16, .04 |  |
| SD | AP | 8.86,  < .001, .16 *** | 0.21,  .65, < .01 | 0.83,  .42, .02 |  |
| SD | ML | 41.26,  < .001, .47 *** | 0.05,  .83, < .01 | 6.55,  .002, .12 ** |  |

Table S9.

Test statistics of repeated-measures t-tests for the target-aligned, log-transformed average (unsigned; AV) and standard deviation (SD) of force moment by direction (AP: anteroposterior; ML: mediolateral) and the current trial congruency

| Moment |  | t, p, dz |
| --- | --- | --- |
| Parameter | Direction | Main effect |
|  |  | Current trial  congruency |
| AV | AP | 0.079,  .94,.01 |
| AV | ML | 1.09,  .28,.16 |
| SD | AP | 0.27,  .79,.04 |
| SD | ML | -1.24,  .22,.18 |

Table S10.

Test statistics for ANOVAs of the response-aligned, log-transformed average (unsigned; AV) and standard deviation (SD) of force moment by direction of body sway (AP: anteroposterior; ML: mediolateral), time bin, and the current trial congruency. Significant effects or interactions are indicated with asterisks.

| Moment |  | F, p, partial eta^2 | | |
| --- | --- | --- | --- | --- |
| Parameter | Direction | Main effect | Main effect | Interaction |
|  |  | Time bin | Current trial  congruency | Time bin by  current trial  congruency |
| AV | AP | .97,  .33, .02 | 1.91,  .17, .04 | 1.49,  .23, .03 |
| AV | ML | 1.04,  .31, .02 | .65,  .43, .01 | .53,  .47, .01 |
| SD | AP | 0.17,  .68, .01 | 2.20,  .14, .04 | 3.02,  .09, .06 |
| SD | ML | 1.76,  .19, .04 | 0.16,  .69, .01 | 8.87,  .005, .16 ** |
